# Cleft palate-lateral synechiae syndrome: a case report

**DOI:** 10.11604/pamj.2022.43.57.33534

**Published:** 2022-10-06

**Authors:** Wafa Id El Mouden, Abdellatif Daoudi, Khalila Nainia, Othmane Benhoummad, Hind Jamil, Houda Majhoul, Samira Tizki

**Affiliations:** 1 Faculty of Medicine and Pharmacy of Agadir, University Ibn Zohr, University Hospital Center of Souss-Massa, Agadir, Morocco,; 2 Department of Pediatrics, University Hospital Center of Souss-Massa, Agadir, Morocco,; 3 Department of Otorhinolaryngology, University Hospital Center of Souss-Massa, Agadir, Morocco

**Keywords:** Cleft palate, intraoral synechiae, intraoral fibrous bands, neonate, case report

## Abstract

Cleft palate-lateral synechiae syndrome (CPLSS) is an extremely rare congenital malformation syndrome with undetermined etiology, characterized by a cleft palate and lateral intraoral synechiae linking the free borders of the palate to the mouth floor. We report a case of a female neonate, admitted for suckling difficulties with a cleft lip and palate associated to multiple lateral intraoral synechiae. Resection of the synechiae allowed oral feeding. Cleft palate-lateral synechiae syndrome is an exceptional syndrome as only seventeen cases have been reported in the literature. Synechiae can be isolated or more frequently in association with other congenital anomalies such as cleft lip and/or palate. These synechiae can cause functional deficits, especially in the respiratory and feeding tracts, language disorders or recurrent otitis. Although it is exceptional, this malformative entity must be known by medical practitioners in order to set up a well-adapted therapeutic protocol.

## Introduction

Intraoral synechiae are cord-like adhesions extending from the inner free edges of the palate to the lateral parts of the tongue or floor of the mouth. They can be asymptomatic or cause eating disorders due to limited opening of the mouth, recurrent otitis or deterioration of language. They may be isolated or most frequently associated with multiple congenital anomalies. Cleft palate-lateral synechiae syndrome (CPLSS) was first described in 1972 [1]; a rare syndrome that includes cleft palate, lateral synechiae and micrognathia. Certain studies have suggested an autosomal dominant inheritance with variable penetrance and expression [2]. The ultimate goal of treatment is to obtain a normal oral opening in order to improve both the airway and feeding, and to permit oral and facial development in a normal manner, as well as surgical treatment of the cleft [2]. The present study reports a new case of cleft lip and palate associated with intraoral synechiae.

## Patient and observation

**Patient information:** a 1-day-old Moroccan newborn female was referred to neonatology team by her pediatrician for a cleft lip and palate with intraoral bands. She was born in 2021 at 41 weeks' gestation by normal vaginal delivery, with a birth weight of 3000 g, to a physically healthy Caucasian G1P1 mother who had no known drug exposure. She was the only child of non consanguineous parents. There was no known family history of orofacial defects or other congenital malformations.

**Figure 1:**
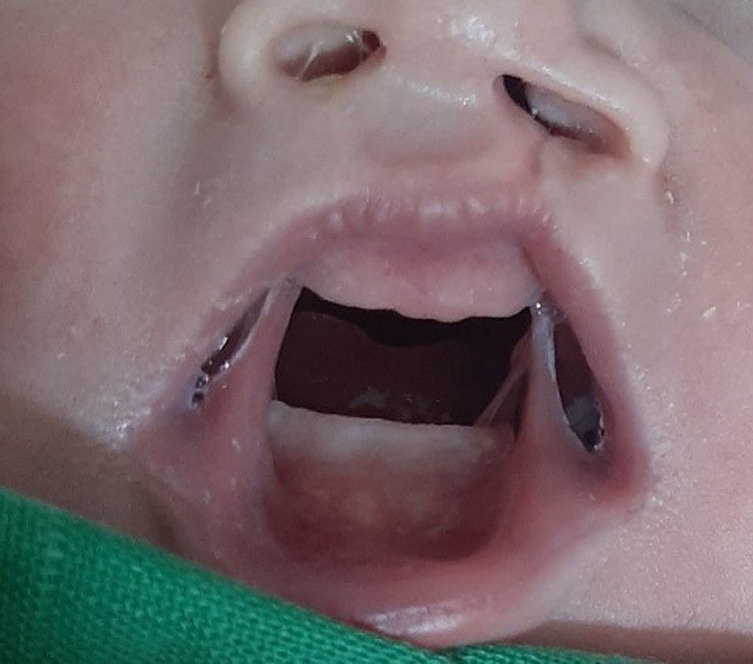
congenital lateral synechiae associated with cleft lip and palate

**Figure 2:**
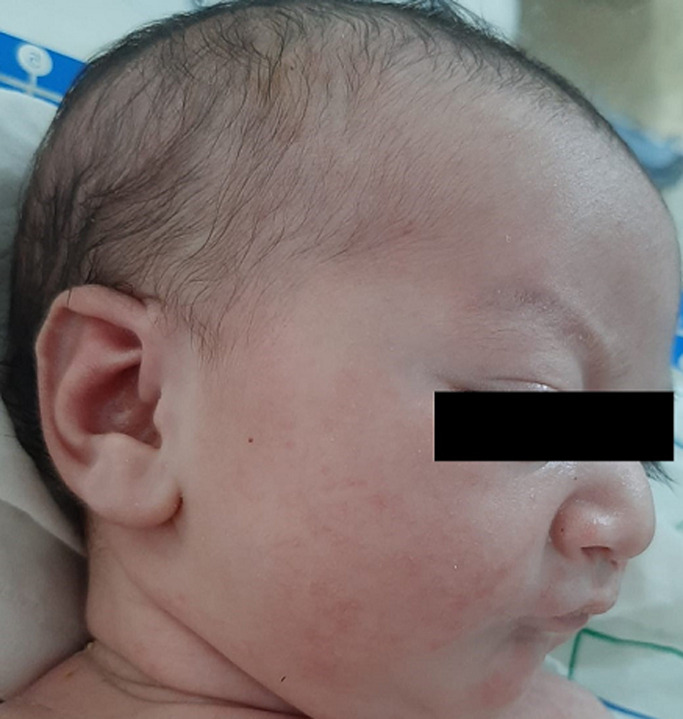
facial features; microretrognathia and prominent nose

**Figure 3:**
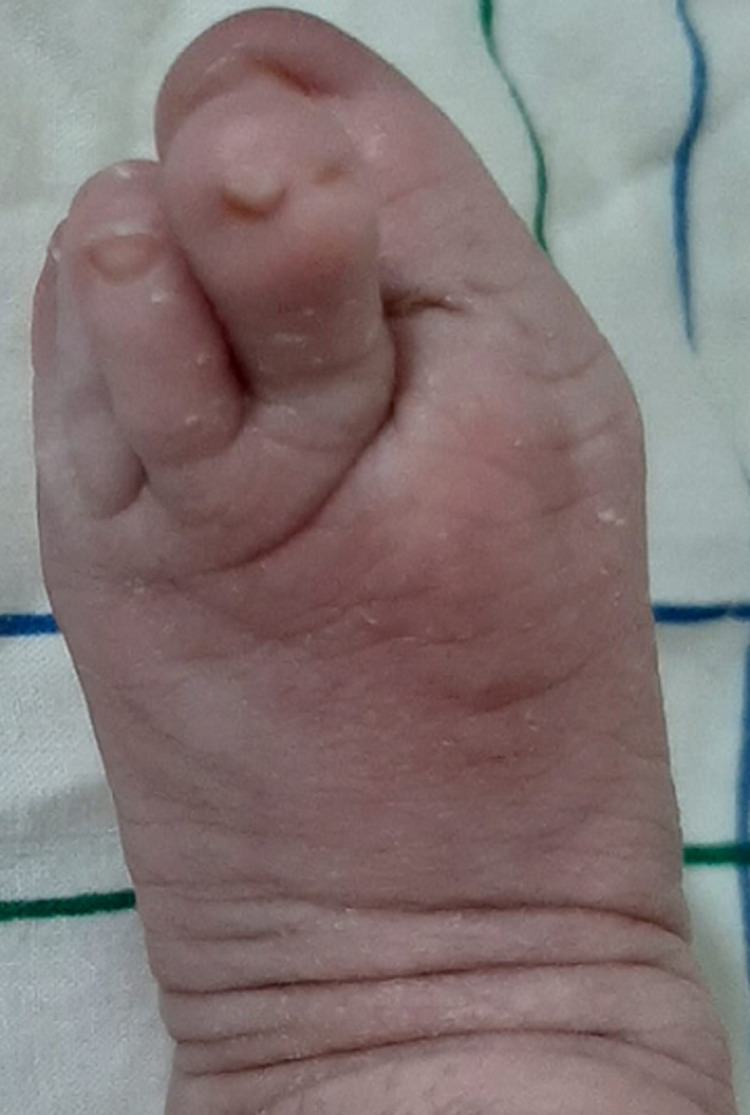
hallux valgus

**Clinical findings:** physical examination at birth objectified a left unilateral cleft lip and palate, bilateral fibrous bands extending between the mandibular and maxillary alveolar ridges (Figure 1), micro-retrognathia (Figure 2), a prominent nose (Figure 2), and left hallux valgus (Figure 3). She had no inferior labial fossae; no musculoskeletal or thoracic abnormalities. There was neither popliteal pterygium nor genital abnormalities.

**Timeline of current episode:** the symptomatology appeared since the birth.

**Diagnostic assessment:** no genetic testing was performed.

**Diagnosis:** the diagnosis of cleft palate-lateral synechiae syndrome was retained in view of the clinical findings.

**Therapeutic interventions, follow-up and outcome of interventions:** at 15 days of age, the neonate was operated under brief general anesthesia administered by a facial mask. The fibrous bands were dissected using bipolar diathermy. This allowed full jaw opening, and normal oral feeding. The postoperative evolution was without incident. The newborn is presently in a good physical health and will continue to be regularly evaluated by the multidisciplinary cleft lip and palate team for further palatoplasty.

**Informed consent:** this study is conforming to the declaration of Helsinki. The patient's parents have provided informed consent regarding the study.

## Discussion

Among the cases of cleft lip and palate described in the literature, isolated cases constitute about 70% of all cases; however intra-alveolar fibrous bands have been rarely reported [2]. Intraoral synechiae are most commonly associated with cleft lip and/or palate and other congenital anomalies as in our case, they are exceptionally isolated. They occur most often in the context of popliteal pterygium syndrome or in association with various congenital malformations of previously unidentified etiology [3].

A new syndrome was first described in 1972 involving a cleft palate associated with multiple fibrotic adhesions extending from the free edges of the palate to the floor buccal cavity [1]. Various terms have been used in the literature to describe these conditions. The term “cleft palate and congenital synechiae syndrome” corresponds to cleft palate-lateral synechiae syndrome (CPLSS) [4]. By contrast, it has been adopted to illustrate the same features as those described as “congenital cleft palate and alveolar synechiae syndrome” [5]. There is a nuance concerning the standard nomenclature. Lateral synechiae (LS) are defined as fibrous bands extending from the cleft edges to the lateral edges of the tongue or floor of oral cavity, whereas alveolar synechiae (AS) are defined as fibrous bands extending between the bi-maxillary alveolar processes [6]. The diagnosis of CPLSS in our case was based on the clinical findings: lateral synechiae in addition to facial abnormalities characteristic of the syndrome were identified. Further syndromes associated with cleft palate and intraoral synechiae have been reported in the literature. Fryns syndrome [7], has been described as a “syndrome of multiple variable congenital abnormalities” [7], including CPLSS in its phenotypic spectrum [3], it comprises musculoskeletal and thoracic malformations. The phenotypic presentation in this case is characterized by AS and not LS [6]. Other syndromes have been reported; in the case of popliteal pterygium syndrome, the synechiae are AS. However, the synechiae identified in Van der Woude syndrome are both LS and AS [8].

To date, the pathophysiological mechanism of intraoral synechiae has not been well demonstrated, but few theories have been suggested based on the local factors interfering during facial development. In particular, the proximity between the palate and the floor of buccal cavity, as well as the tongue interposed between the palatal tablets, which constitutes a favorable condition for the development of synechiae. The theory that explains the occurrence of synechiae as a result of pressure on the first bronchial arch due to ischemic amniotic bands, or as a consequence of the persistence of the buccopharyngeal membrane has been also suggested [4].

No impact on fetal development due to synechiae has been mentioned in the literature; however feeding difficulties have been mentioned in some cases as well as the clinical impact of lateral synechiae on newborns [3,9]. In the majority of reported cases, synechiae were resected previous to palatoplasty a few days to a few weeks after birth, except for three cases with untreated synechiae until childhood [10]. However, these patients tolerated LS and did not present any problems concerning the mouth opening or feeding [10]. In our case, a regular follow-up of the patient was realized during 2 months, there was no complication mentioned, mainly no difficulty in feeding or breathing, and the baby kept a normal mouth opening. Further research is warranted in this context to better understand the pathophysiology and consequently establish an appropriate management for each patient.

## Conclusion

Congenital intra-oral fibrous bands are rarely reported in the literature, but their repercussion is mainly functional, especially the respiratory and feeding pathway. They can occur in an isolated way or more frequently in a syndromic context as in our case. An early and adequate management facilitates the oral opening as well as the correction of the associated malformations.

## References

[1] Fuhrmann W, Koch F, Schweckendiek W (1972). Autosomal dominant inheritance of cleft palate and synechias between the palate and floor of the mouth or tongue. Humangenetik.

[2] Vamvanij N, Chen ZC, Lo LJ (2020). Patients with cleft lip and palate associated with intraoral fibrous bands: a report of 3 cases and review of literature. Cleft Palate Craniofac J.

[3] Jaeger A, Kapur R, Whelan M, Leung E, Cunningham M (2003). Cleft-palate lateral synechia syndrome: insight into the phenotypic spectrum of Fryns syndrome. Birth Defects Res A Clin Mol Teratol.

[4] Murphy SM, Rea S, McGovern E, Fleming P, Orr D (2004). Cleft palate and congenital synechiae syndrome: a case report. Cleft Palate Craniofac J.

[5] Dalal M, Davison PM (2002). Cleft palate congenital alveolar synechiae syndrome: case reports and review. Br J Plast Surg.

[6] Imai Y, Tachi M (2020). Congenital lateral palatal synechia associated with cleft palate: a case report with long-term follow-up and review of the lkiterature. Cleft Palate Craniofac J.

[7] Fryns JP, Moerman F, Goddeeris P, Bossuyt C, Van den Berghe H (1979). A new lethal syndrome with cloudy corneae, diaphragmatic defects and distal limb deformities. Hum Genet.

[8] Robbins A, Zarate YA, Hartzell LD (2019). Combined tongue-palate fusion with alveolar bands in a patient with Pierre Robin sequence and Van der Woude syndrome. Cleft Palate Craniofac J.

[9] Sybil D, Sagtani A (2013). Cleft palate lateral synechia syndrome. Natl J Maxillofac Surg.

[10] Nakata NM, Guion-Almeida ML, Richieri-Costa R (1993). Cleft palate-lateral synechiae syndrome: report on three new patients with additional findings and evidence for variability and heterogeneity. Am J Med Genet.

